# Collateral pulmonary vein after catheter ablation therapy for atrial fibrillation

**DOI:** 10.1259/bjrcr.20200184

**Published:** 2021-07-08

**Authors:** Kyoko Nagai, Akio Kotake, Yoshiro Hori, Nobuyuki Takeyama, Eliko Tanaka, Yuki Tashiro, Toshi Hashimoto, Daisuke Wakatsuki, Hiroshi Suzuki

**Affiliations:** 1 Department of Radiology, Showa University Fujigaoka Hospital, Yokohama, Japan; 2 The Cardiovascular Division of Internal Medicine, Showa University Fujigaoka Hospital, Yokohama, Japan; 3 The Cardiovascular Division of Internal Medicine, Showa University Fujigaoka Hospital, Yokohama, Japan

## Abstract

A patient with previous catheter ablation therapy for atrial fibrillation was examined for an abnormal shadow on a chest radiograph. ECG-gated multidetector CT clearly showed the left upper pulmonary vein connected with the left inferior pulmonary vein. We hypothesize an intrapulmonary venous connection as a collateral.

## Case presentation

**Figure 1.**
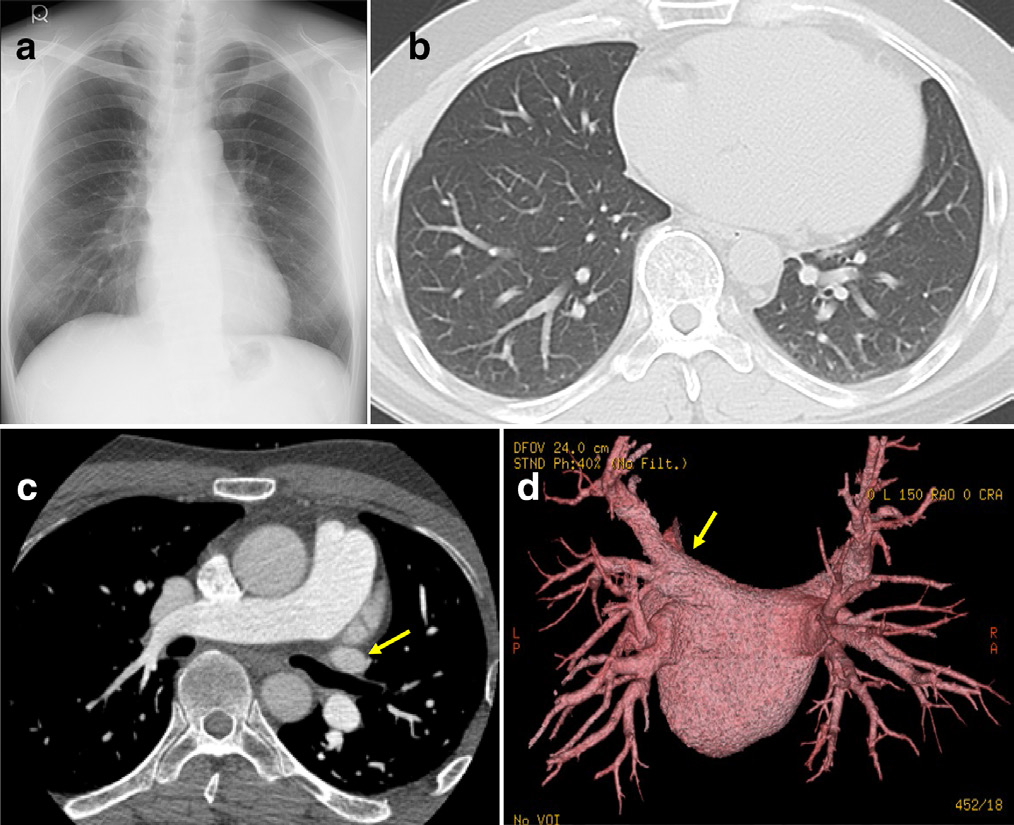
Before catheter ablation therapy (**A**) Chest radiograph demonstrated no abnormality before catheter ablation therapy. (**B**) CT showed no consolidation and no serpiginous vascular abnormality in the lingular segment LUL. (**C**) ECG-gated multidetector CT revealed no stenosis of the left upper PV (arrow). (**D**) Three-dimensional volume-rendered color-coded CT angiographic posterior image showed no PV stenosis. The left upper PV (arrow). LU, left upper lobe; PV, pulmonary vein.

A 44-year-old asymptomatic Japanese male received catheter ablation therapy twice after suffering from atrial fibrillation (AF). The first ablation therapy was performed 3 years ago, while the second was performed 2 years, 3 months after. There was no stenosis of any pulmonary vein (PV) and lung field abnormalities before the first catheter ablation therapy (Figure 1). The patient had no medical history except for catheter ablation therapy for AF.

## Investigations

**Figure 2.**
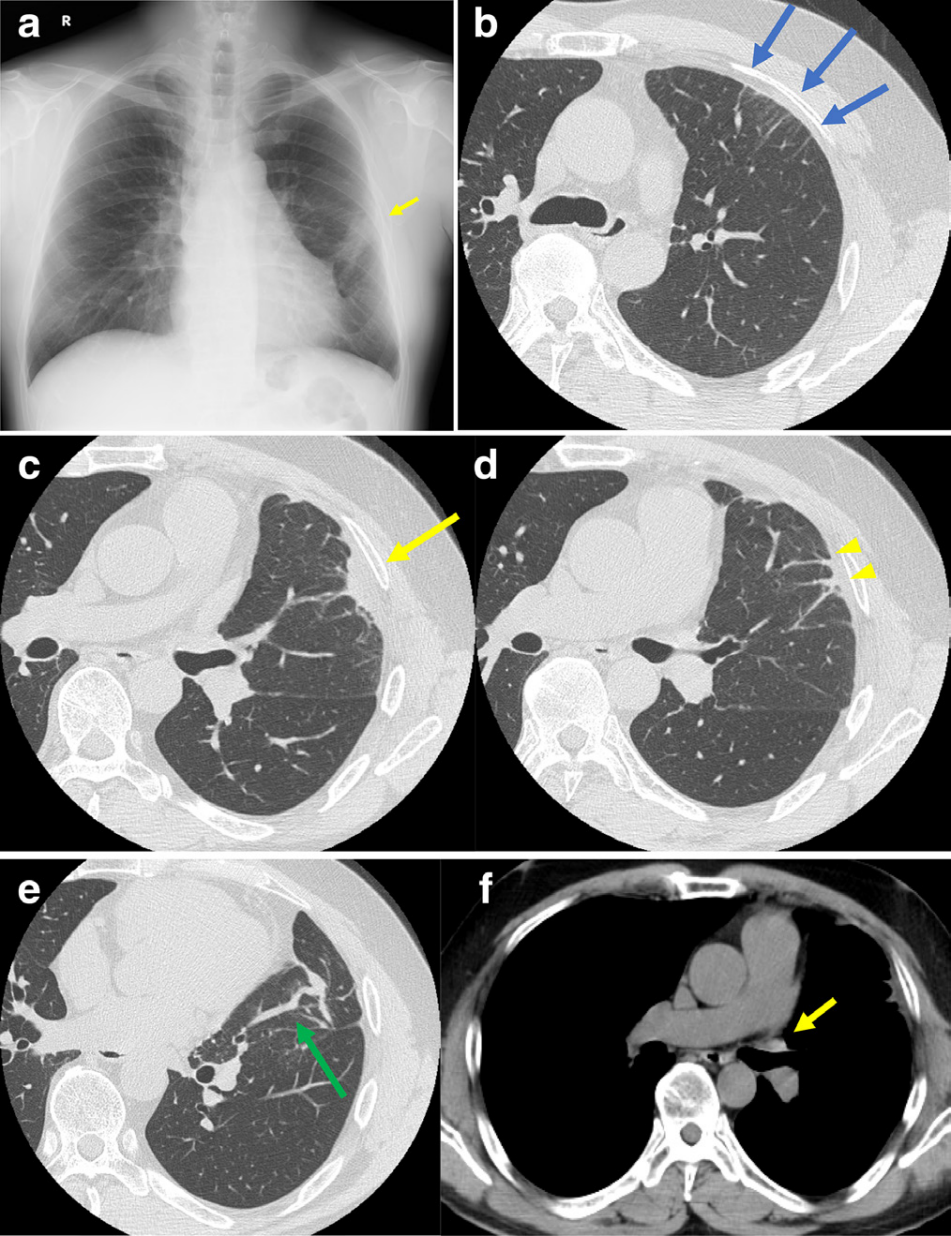
1 year and 3 months after second catheter ablation therapy (**A**) Chest radiograph demonstrated the consolidation in the left middle lung zone (**B**–**E**) CT revealed linear opacities (blue arrow), parenchymal bands (yellow arrow head), peripheral consolidation (yellow arrow) and the dilatated and tortuous pulmonary vein (green arrow) in the anterior and lingular segment of the LUL. The left major fissure was incomplete lobulation. (**F**) Plain CT images brought the stenosis of the left upper PV (yellow arrow) near the atrial junction. LUL, left upper lobe; PV, pulmonary vein.

He was followed up at the outpatient department and no AF recurrence was observed. Chest radiography showed pulmonary consolidation in the left middle lung (Figure 2a) at a routine medical checkup, 1 year, 3 months after the second catheter ablation therapy.

He complained of cough and hemoptysis 1 week later. Non-enhanced CT showed linear opacities, parenchymal bands, and peripheral consolidation in the anterior and lingular segment of the left upper lobe (LUL) (Figure 2b–d). Pneumonia was initially suspected.

Retrospective CT analysis showed that the stenosis of the left upper PV appeared near the atrial junction and the dilatated and tortuous PV in the lingular segment of the LUL (Figure 2e–f). The left major fissure was incomplete lobulation (Figure 2b–f). PV stenosis and venous infarction after ablation therapy for AF were diagnosed.

**Figure 3.**
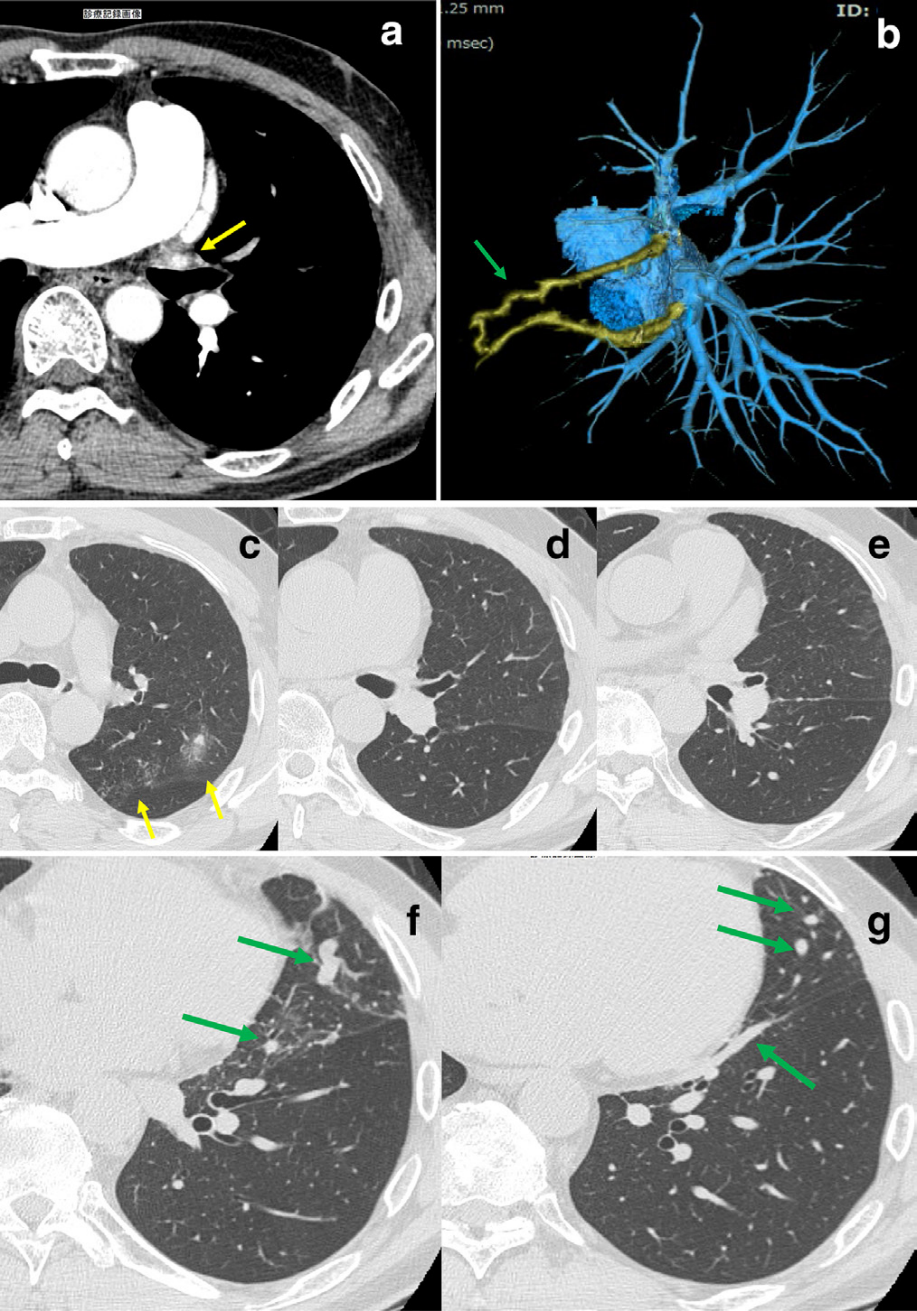
ECG-gated multidetector CT, 3 years and 11 months after second catheter ablation therapy (**A**) Enhanced CT revealed the left upper PV stenosis (yellow arrow). (**B**) Three-dimensional volume-rendered color-coded CT angiographic left lateral image showed the anomalous tortuous vein (green arrow) between the upper PV and lower PV. (**C**–**G**) The anomalous tortuous vein (green arrow) began from V4, ran caudally along the interlobar pleural surface and finally drained into the lower PV. Nodular ground glass opacities (yellow arrow) newly appeared at the apicoposterior segment of the LUL. However, the peripheral opacities and linear opacities in the LUL disappeared. The left lung field showed incomplete lobulation. LUL, left upper lobe; PV, pulmonary vein (Supplementary Videos 1 and 2 about is available at BJR case reports online).

He underwent ECG-gated multidetector CT 3 years, 11 months after the second catheter ablation therapy. CT revealed left upper PV stenosis and an anomalous intrapulmonary venous connection between the left upper PV and the left lower PV. The anomalous tortuous vein began from V4, ran caudally along the interlobar pleural surface and finally drained into the lower PV. Nodular ground glass opacities newly appeared at the apicoposterior segment of the LUL. However, the peripheral opacities and interstitial septal thickening in the LUL disappeared (Figure 3).

Supplementary Video 1.

Supplementary Video 2.

The patient was diagnosed with collateral pulmonary vein secondary to PV stenosis after catheter ablation therapy for AF.

## Differential diagnosis

The initial differential diagnosis for peripheral consolidation after catheter ablation therapy was pulmonary venous infarction, alveolar hemorrhage and nonspecific pneumonia.^1,2^ The differential diagnosis for anomalous intrapulmonary venous connection was pulmonary arteriovenous malformation, hypogenic lung (Scimitar) syndrome, an anomalous unilateral single PV (the term *meandering PV* has also been used to refer to this condition), and collateral pulmonary vein.^3^

## Outcome and follow-up

The patient has remained well at subsequent clinic follow-up.

## Discussion

It is well-known that PV stenosis and pulmonary venous hypertension or venous infarction are relatively uncommon complications of radiofrequency ablation therapy for AF.^1,2^

An anomalous intrapulmonary venous connection is rare after acquired PV stenosis.^4^

In the present study, ECG-gated multidetector CT clearly revealed left upper PV stenosis and an anomalous intrapulmonary venous connection. An anomalous intrapulmonary venous connection was distinguished from pulmonary arteriovenous malformation and hypogenic lung syndrome (Scimitar syndrome). Arteriovenous malformation and hypogenic lung syndrome may require surgery or embolization to correct the shunt. In contrast, an anomalous intrapulmonary venous connection has no vascular shunt. This case was managed conservatively because the patient presented with mild symptoms such as hemoptysis and cough once and then became asymptomatic. An anomalous unilateral single PV is a rare congenital pulmonary venous abnormality where a single vein enters ipsilaterally into the left atrium after receiving from all the PVs.^5^ An anomalous unilateral single PV requires no treatment because no vascular shunt is produced.^5,6^

Lung parenchyma showed interstitial septal thickening, parenchymal bands, peripheral consolidation in the anterior and lingular segment of the LUL was considered pulmonary venous hypertension, venous infarction and alveolar hemorrhage,^1,2^ 1 year and 3 months after the second catheter ablation therapy (Figure 2).

**Figure 4.**
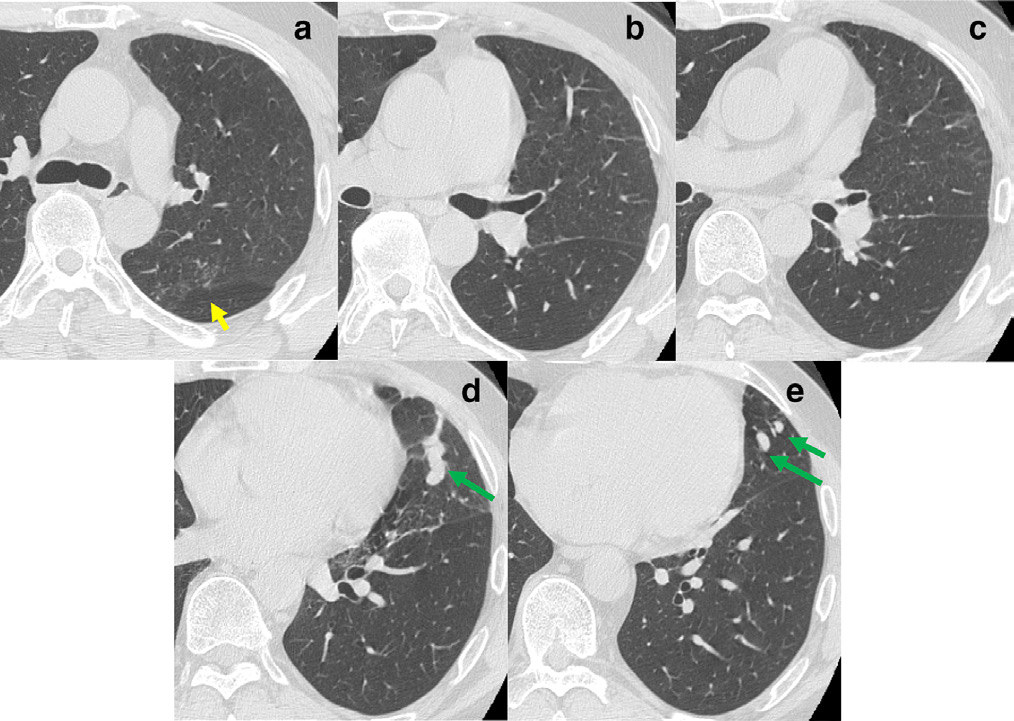
(A)-(E) The nodular ground glass opacities (yellow arrow) at the apical posteriorsegment LUL were unclear and the meandering PV (green arrow) was more obvious.

The newly appeared nodular ground glass opacities at the apicoposterior segment LUL, including the peripheral opacities and interstitial septal thickening in the LUL, disappeared, 3 years, 11 months after the second catheter ablation therapy (Figure 3). A follow-up CT 5 years after the second catheter ablation therapy confirmed an almost complete resolution of the lung parenchymal abnormalities and the anomalous pulmonary venous connection was more obvious (Figure 4). The CT findings of the lung parenchyma and the anomalous PV changed over time, and the anomalous tortuous vein may compensate for intrapulmonary venous drainage.

Anatomically, PVs and bronchial veins are interconnected through already existing bronchial venous plexuses. These plexuses are located in the bronchial wall and the peribronchovascular connective tissues.^7^ In this case, left upper PV stenosis was the cause of high PV pressure in the LUL. We speculated that left upper PV and the bronchial venous plexuses were interconnected, while bronchial venous plexuses and the other left lower PV were also interconnected. The PV stenosis gradually led to an anomalous intrapulmonary venous connection as an intrapulmonary collateral.

A collateral pulmonary vein after catheter ablation therapy for atrial fibrillation is rare. Recognition of this condition is important. ECG-gated multidetector CT can clearly reveal an acquired anomalous intrapulmonary venous connection; therefore, we should prevent the use of invasive diagnostic and therapeutic procedures.

## Learning points

1. The presented case illustrates the emergence of an anomalous intrapulmonary venous connection after catheter ablation therapy.
2. Little is known about a collateral pulmonary vein after catheter ablation therapy, but recognition of this condition is important. The finding of a venous collateral has no vascular shunt, therefore, we should prevent the use of invasive diagnostic and therapeutic procedures.
3. The differential diagnosis for anomalous intrapulmonary venous connection was pulmonary arteriovenous malformation, hypogenic lung (Scimitar) syndrome, an anomalous unilateral single PV (the term *meandering PV* has also been used to refer to this condition), and collateral pulmonary vein.
